# Synthetic strategies of supported atomic clusters for heterogeneous catalysis

**DOI:** 10.1038/s41467-020-19571-6

**Published:** 2020-11-18

**Authors:** Hongpan Rong, Shufang Ji, Jiatao Zhang, Dingsheng Wang, Yadong Li

**Affiliations:** 1 Beijing Key Laboratory of Construction-Tailorable Advanced Functional Materials and Green Applications, School of Materials Science and Engineering, Beijing Institute of Technology, Beijing, 100081 China; 2 Department of Chemistry, Tsinghua University, Beijing, 100084 China

**Keywords:** Catalyst synthesis, Heterogeneous catalysis, Synthesis and processing

## Abstract

Supported atomic clusters with uniform metal sites and definite low-nuclearity are intermediate states between single-atom catalysts (SACs) and nanoparticles in size. Benefiting from the presence of metal–metal bonds, supported atomic clusters can trigger synergistic effects among every metal atom, which contributes to achieving unique catalytic properties different from SACs and nanoparticles. However, the scalable and precise synthesis and atomic-level insights into the structure–properties relationship of supported atomic clusters is a great challenge. This perspective presents the latest progress of the synthesis of supported atomic clusters, highlights how the structure affects catalytic properties, and discusses the limitations as well as prospects.

## Abstract (web summary)

Supported atomic clusters with precise nuclearity are intermediate states between single-atom catalysts and nanoparticles in size. Here the authors summarize and discuss synthetic strategies of supported atomic clusters with unique catalytic properties for heterogeneous reactions.

## Introduction

Heterogeneous catalysts are widely used in industrial processes because they are easy to separate and recycle. Traditional heterogeneous nanocatalysts are usually prepared by impregnation or coprecipitation methods, resulting in a polydispersity in the number of atoms: nanoparticles, clusters, and even single atoms^1^. The size of metal species determines the fraction of surface atoms, which has a significant effect on both catalytic activity and selectivity. What is more, in the subnanometer size regime, the addition or removal of only one atom may bring about substantial influences on their catalytic properties^2–6^. Therefore, the traditional heterogeneous nanocatalysts are like a “black box”, which makes the characterization and identification of active metal species a significant challenge. Furthermore, metal species with different sizes or structures may affect the adsorption and activation of substrates, triggering side reactions, thus decreasing the selectivity of catalysts.

The crucial first step to address these problems effectively is preparing heterogeneous catalysts with monodisperse metal species. Second, we have to identify the real active sites of catalysts systematically in the reaction process. Finally, a deep understanding of the relationship between their structures and properties presents a promising framework for the design and optimization of catalysts at the atomic scale.

Stemming from the unique geometric and electronic structures, supported atomic clusters with metal–metal bond and definite low-nuclearity metal active centers (especially the nuclearity of metallic atoms <10) can exhibit distinct catalytic properties^1,7–9^. In supported atomic clusters, most of the metal atoms are exposed as much as possible and are available for the reactant molecules. Therefore, supported atomic clusters have much more atom utilization efficiency in catalytic reactions than corresponding nanoparticles. In addition, due to the nanoparticles with the large size and complicated structure, the reactant molecules will be absorbed on uncertain sites of the surface of the nanoparticles, such as edge sites, corner sites, and planes, and formed different geometric structures with nanoparticles, resulting in a low catalytic selectivity. The investigation about supported atomic clusters with limited nuclearity provides a potential opportunity to reveal the real active sites in catalysis.

**Fig. 1:**
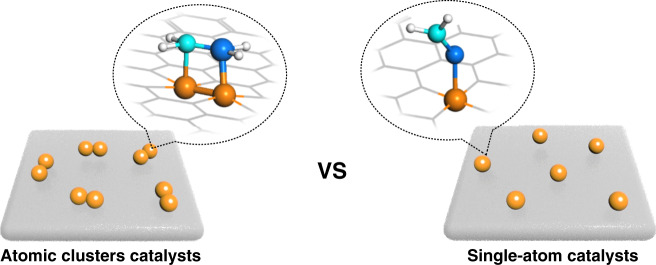
Comparison diagram of adsorption modes in catalysis. Schematic view and corresponding structure model of the supported atomic clusters catalysts (left) and the single-atom catalysts (right).

With mononuclear metal centers, single-atom catalysts (SACs) have drawn a broad interest in the field of catalysis. Many SACs have been synthesized by strategies including pyrolysis, external field-assisted method, ball milling, photochemical reduction, atomic layer deposition, etc^10,11^. With 100% atom utilization efficiency and strong metal–support interaction, SACs have shown outstanding performance in many chemical conversions, including thermochemical, electrochemical, and photochemical reactions^12–15^. However, in many critical industrial processes, especially in the catalytic reactions of multiple substrates, the single atomic active site cannot adsorb and activate multiple substrates at the same time, thus reducing the catalytic efficiency or even causing inactivity for the reaction (see Fig. 1)^16^. In contrast, supported atomic clusters with two or more atoms can provide enough sites for the adsorption and activation of multiple substrates, which may endow a new catalytic pathway, decreasing the reaction barrier and improving the catalytic activity^17^. For example, for the selective oxidation reaction of alcohols to aldehydes, the calculated turnover frequency (TOF) of Ru_3_/CN is more than ten times higher than that of Ru_1_/CN^18^. Compared with SACs, supported atomic clusters not only provide more activation sites in each isolated catalytic site but also have different electronic structures due to the orbital overlapping between metal atoms. The synergistic effect among metal atoms for boosting catalytic performances is uniquely distinct in supported atomic clusters that contain two or more kinds of metal atoms. For instance, in terms of acid electrochemical oxygen reduction reaction (ORR), dual Fe–Co sites supported on N-doped porous carbon exhibit higher activity than both Fe- and Co-SACs^19^. With the development of synthesis and characterization, supported atomic clusters will undoubtedly be the next key and hot topic.

**Fig. 2:**
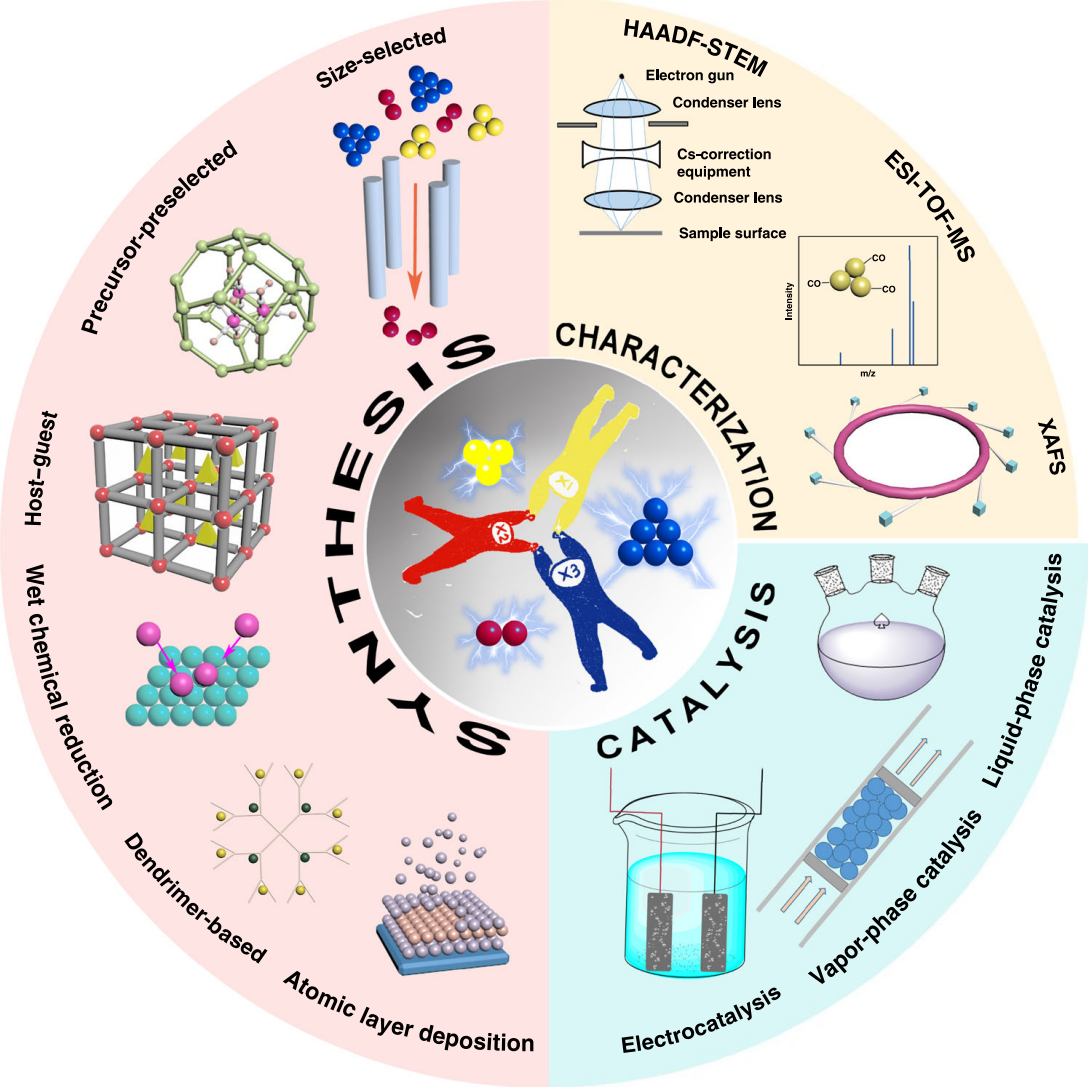
Synthesis and catalytic performances of supported atomic clusters. Schematics for the size-selected, precursor-preselected, host–guest, wet chemical reduction, dendrimer-based, and atomic layer deposition strategies of supported atomic clusters; HAADF-STEM, ESI-TOF-MS, and XAFS are three important and typical characterization techniques; the electrochemical, vapor-, and liquid-phase catalytic performances of supported atomic clusters.

With recent developments in both theoretical modeling and advanced atomic-resolution characterization technology, the concept of single-cluster catalysts has been popular in the past few years. Combing the atomic-resolution technologies and calculations, researchers can provide a clear image of the dispersion and geometry of the clusters, even their exact positions, and detailed local coordination environment. Unfortunately, the controlled synthesis of supported atomic clusters is considerably complicated because it is difficult to stabilize the precise numbers of atoms on the support rationally. As shown in Fig. 2, several synthetic strategies have been proven effective. In a long history, gas-phase synthesis and size-selected strategy was the only method to prepare supported metal clusters with precise numbers of atoms^20–25^. However, the throughput of this method is only enough for analytical-scale study and far from the requirement of the catalytic applications. Then, new strategies that can afford large-scale metal clusters were proposed. These synthetic methods include precursor-preselected strategy^18,26,27^, host–guest strategy^19,28,29^, wet chemical reduction^30,31^, dendrimer-based^32,33^, and atomic layer deposition method^34^. Considerable scientists have focused on the support effects and dynamic behavior of supported atomic clusters during heterogeneous catalytic processes via in situ techniques and theoretical calculations^35–38^. Apart from synthesis and dynamism, extensive efforts have also been devoted to predicting the catalytic properties of supported atomic clusters by computational studies^5,39–41^. For example, first-principle theoretical studies show that Fe_3_/θ-Al_2_O_3_(010) and Rh_1_Co_3_/CoO(011) can catalyze the thermal conversion of N_2_ to NH_3_ via an associative mechanism^42,43^. The research of supported atomic clusters is in an eruption period.

In this perspective, we provide recent achievements in the synthesis of supported atomic clusters and discuss their structure-properties relationship from the atomic-level insights. Then we outline the prospects and challenges for the synthesis, characterization, and catalysis of supported atomic clusters.

## The size-selected strategy

**Fig. 3:**
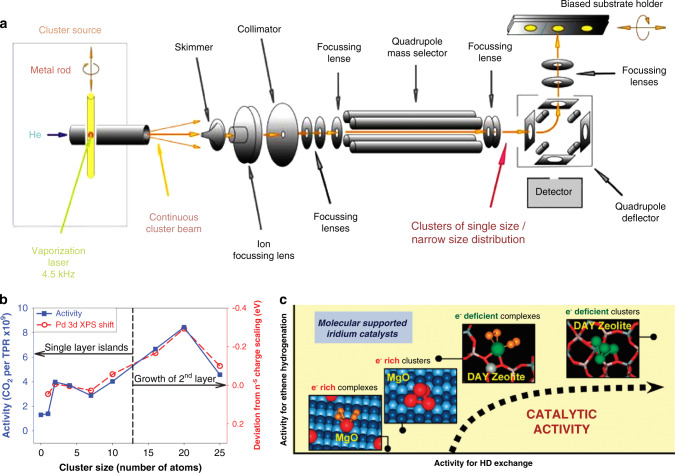
The size-selected strategy. **a** Schematic of the cluster deposition setup, reprinted with permission from Springer Science+Business Media, Inc.: Springer Nature, *Topics in Catalysis*, ref. ^44^ (Winans et al.). Copyright (2006). **b** CO oxidation activity observed during the temperature-programmed reaction (left axis, solid squares) compared with shifts in the Pd 3d binding energy, relative to expectations from smooth bulk scaling (right axis, open circles), as a function of cluster size. From ref. ^7^. Reprinted with permission from the American Association for the Advancement of Science (AAAS). **c** Schematic illustration of mononuclear Ir and Ir_4_ clusters supported on MgO and dealuminated HY zeolite. Their relative catalytic activities for ethane hydrogenation are also presented, reprinted with permission from ref. ^71^. Copyright (2011) American Chemical Society.

As noted above, the gas-phase synthesis and size-selected method have a long history, and numerous metal clusters with precise numbers of atoms have been prepared through this approach. Three steps, including the creation, separation, and landing of clusters, form the whole process. For the cluster generation, different techniques including laser vaporization (see Fig. 3a)^44^, magnetron sputtering^45^, cold or hot reflex discharge ion source^46^, arc discharge^47^, and electrospray ionization^48^ have always been used. However, clusters produced by these sources usually exhibit a wide distribution of sizes. To obtain charged metal clusters with well-defined composition and precise size, researchers have to do the size-selection process. Commonly utilized routes for size-selection of clusters are electrostatic quadrupole mass filters^49^, radiofrequency quadrupole mass filters^50^, and time of flight^51^. To prevent the cluster fragmentation and substrate damage, researchers developed the landing technique from “hard landing” to “soft landing”^52,53^. Utilizing inert gas, or opposite electric field, the kinetic energy of the cluster can be decreased, and monodisperse samples can be produced^21^. Not only monometallic clusters, but this approach can also form binary alloy clusters^54^. Comprehensive reviews have been written comparing the advantages and disadvantages of each size-selection route in different catalytic applications^37,55–57^.

Due to its simplicity and significance, CO oxidation has been widely investigated^1,7^. Because of the high activity in the CO oxidation, in particular, for the low-temperature conditions, Au-based supported atomic clusters have attracted lots of attention. Compared with the Au SACs, Au supported atomic clusters showed higher activity in most cases^58,59^. In 2004, Anderson et al. studied the activity of soft-landed Au_n_ clusters (*n* = 1, 2, 3, 4, and 7) on TiO_2_ for CO oxidation. They observed that Au_3_ showed substantial activity, while Au_1_ and Au_2_ did not catalyze CO oxidation^20^. Size-dependent catalytic activity for CO oxidation has also been demonstrated over size-selected Pt_n_ and Pd_n_ supported atomic clusters^60^. The significant support effect on the catalytic performance should also be taken into account. Brune et al. studied the impact of the reduction state of the support on the CO oxidation over Pt_7_/TiO_2_^61^. The Pt_7_ clusters supported on highly reduced TiO_2_ showed the activity of two orders of magnitude lower than on the slightly reduced one. The decreased activity resulted from the oxygen consumption by the surface segregation of Ti^3+^ interstitials. However, comparisons were not made over supported atomic clusters with SACs in these reports. According to the report of Anderson et al.^7^, there is a good correlation between the catalytic activity and the Pd 3d X-ray photoelectron spectroscopy (XPS) shift, indicating the electronic structure of Pd_n_ supported atomic clusters has a significant effect on the catalytic performances (see Fig. 3b). The Pd 3d binding energy varied non-monotonically with nuclearity number of supported atomic clusters. However, a stable valence electronic structure of Pd supported atomic clusters showed high 3d binding energy, leading to low CO oxidation activity. By using He^+^ ion scattering, Anderson et al. also found that the single-to-double layer geometric transition of the clusters was parallel with the change of catalytic activity. The geometric transition has also been observed in the cases of Au_n_ and Pt_n_ clusters in a similar nuclearity number range (about 10)^1,8^. For the active sites of Pd_n_ supported atomic clusters for CO oxidation, the debate focuses on whether oxygen should be contained in the sites or not. Considerable experimental and theoretical results have supported the vital role of oxygen for the CO oxidation, especially when the host is the Ce-based materials^62–65^. When the support is amorphous Al_2_O_3_, Pt_10_, and Ag_9_Pt_2,3_ clusters exhibit turnover rate (TOR) of 3350 and 2500 CO_2_ molecules/cluster/s at 300 °C, respectively. The activation energies of Ag_9_Pt_2,3_ and Pt_10_ are around 50 and 60 kJ/mol, respectively^66,67^. By comparison, the apparent activation energies of Au clusters are between 25 and 30 kJ/mol. Although Pt_n_ and Pd_n_ supported atomic clusters have exhibited high performance for CO oxidation, they can hardly surpass Au catalysts. Electronic (the energy level of the HOMO) and the geometric (the number of the layer) structure transition of all the clusters (Au_n_, Pt_n_, and Pd_n_) determined the catalytic activity of CO oxidation.

The dehydrogenation of alkanes, exceptionally light alkanes, to olefins is essential in the chemical industry. Since the reaction is endothermic, the process needs intense energy. Anderson et al. studied the size-selected Pt_n_/Al_2_O_3_ (*n* = 4, 7, and 8) for the dehydrogenation of ethylene to acetylene. With similar nuclearity numbers, Pt_7_ and Pt_8_ supported atomic clusters showed significant catalytic differences in this reaction, and Pt_7_ were more active than Pt_8_ and Pt_4_ supported atomic clusters. The geometric transition from single- to multi-layer, accompanied by changes of charge transfer and binding sites, accounts for the drop between Pt_7_ and Pt_8_ supported atomic clusters^5^. The reactions from olefins to epoxides are very crucial to the production of monomers in the polymer industry. Au- and Ag-based catalysts are always used for selective epoxidation of olefins^68^. The production of useless CO_2_ has long been the problem in the propene epoxidation catalyzed by conventional Ag catalysts. Vajda et al. reported that the size-selected Ag_3_/Al_2_O_3_ supported atomic clusters could efficiently catalyze this reaction with a negligible amount of CO_2_ generation at a low temperature. Although higher temperature would cause the aggregation of Ag_3_ atomic clusters into Ag nanoparticles (NPs, ca. 3.5 nm), the formed NPs exhibited enhanced selectivity towards propene oxide at temperatures lower than 120 °C while maintaining a comparable activity to that of Ag_3_ supported atomic clusters^23^.

The hydrogenation of olefins is a well-known structure-insensitive reaction for platinum group metal nanocatalysts. However, when the size of the catalyst decreased to the sub-nanometric level, the structure-insensitive reaction changes to be structure-sensitive. Pérez-Ramírez et al. showed an inverse correlation between the catalytic activities and the degree of oxidation of Pd species for the semi-hydrogenation of 2-methyl-3-butyn-2-ol^69,70^. Gates et al. studied the catalytic performance of Ir_1_ SACs and Ir_4_ supported atomic clusters on different kinds of supports (γ-Al_2_O_3_, MgO, and zeolite) for the hydrogenation of olefins^71,72^. Due to the new electronic structure/propensities of Ir_4_ clusters, which are not possessed by isolated atoms, the supported Ir_4_ atomic clusters showed higher catalytic activity than Ir_1_ SACs when the supports are the same. Further, they demonstrated that the extent of the nuclearity influence of the Ir species is closely related to the nature of supports. The activity gap between Ir_1_ and Ir_4_ species supported on MgO is much higher than that on zeolite. Besides, when the metal nuclearity was the same, the Ir_4_ atomic clusters supported on zeolite and γ-Al_2_O_3_ showed higher catalytic activity than Ir_4_ atomic clusters supported on the MgO (see Fig. 3c). Since MgO was more electron-donating than zeolite and γ-Al_2_O_3_, Ir sites were electron-rich when they were supported on it; the electron enrichment of Ir sites reduced the capacity of Ir to react with and form ligands from C_2_H_4_ and H_2_, hindering the activation of H_2_. Accordingly, the catalytic performances of metal atomic clusters can be significantly tuned by the nature of metal–support interactions.

Since the gas-phase synthesis and size-selected method, the only method to prepare supported atomic clusters in a long time, require ultrahigh-vacuum (UHV) conditions, the vast majority of catalytic researches of supported atomic clusters have focused on reactions under UHV conditions, which is far away from the realistic and industrial applications^37^. In the cases introduced above, scientists did these model catalytic reactions under UHV by adding a small volume of reaction gases. The catalytic mechanism and significant influence from cluster size and support effect on catalytic performance have been revealed. However, it is still a challenge to apply the synthesized atomic clusters catalysts to the actual catalytic reaction conditions to meet the industrial demand. Researchers have recently gained a series of achievements in studying the catalytic performances of supported atomic clusters under realistic conditions. Anderson et al. have studied atomic clusters supported on glassy carbon or indium tin oxide in UHV, and transferred them to an antechamber without air exposure. In this antechamber, the electrocatalytic performances of supported atomic clusters for oxygen reduction or ethanol oxidation can be explored^73^. Vajda et al. have focused on UHV and in situ reaction conditions of heterogeneous catalytic reactions, including dehydrogenation, epoxidation, and Fischer–Tropsch synthesis^74,75^. It is noteworthy that the gap between ideal UHV and realistic reaction conditions is not easy to be addressed. Some unexpected changes, such as the oxidation state of the cluster, reconstructions of support and clusters, and contaminants, may happen at increased pressures and temperatures. Therefore, it is a great challenge to construct a link between ideal UHV and realistic reaction conditions. Some efforts have been made to try to solve this problem. For example, Vajda et al. prepared two kinds of support for the size-selected Pt_10_ clusters: (1) a highly ordered alumina obtained under UHV, (2) amorphous alumina deposited on a silicon chip that is a similar model of real-world supports. They found both the Pt_10_ clusters and the support changed when Pt_10_/UHV-alumina system was exposed to the realistic reaction conditions. The Pt_10_ clusters transformed from flat, two-dimensional configurations into three-dimensional configurations, and the ordered UHV-alumina was found to transform into an amorphous state^67^.

In a word, the size-selected method is effective to prepare monometallic atomic clusters with precise nuclearity; bimetallic and multimetallic clusters can also be prepared. However, the sophisticated equipment required for this method and the limited output are the major drawbacks.

## The precursor-preselected strategy

The sintering of metal species can be described by the Ostwald ripening process, whose driving force is the different surface diffusion energy of metal atoms with different sizes. As a result, the sintering can be significantly suppressed if the size distribution of metal species is narrow. To prevent sintering during the synthesis of supported atomic clusters, the precursor-preselected strategy is advantageous as the metal species is homogeneous from the beginning to the end. The key to precursor-preselected strategy is the selection of metal precursors to obtain corresponding supported atomic clusters. For example, to prepare M_n_ atomic clusters, we need to select or synthesize atom-precise, ligand stabilized metal molecule, or supramolecule [M_n_L_m_] as the precursor. Due to the diversity and the relatively easy removal of CO, metallic cluster carbonyl had become the preferred precursor. In the 1970s and 1980s, scientists used metal cluster carbonyls as the precursor to prepare surface-supported metal clusters by decarbonylation at elevated temperatures^26,76–82^. Without atomic-resolution technologies, Infrared spectroscopy was a powerful characterization method to identify the structure of atomic clusters in these early studies. Although it was indicated that this approach could fabricate highly dispersed metal species, it was clear that the decarbonylation procedure is inevitably accompanied by cluster aggregation^79^.

**Fig. 4:**
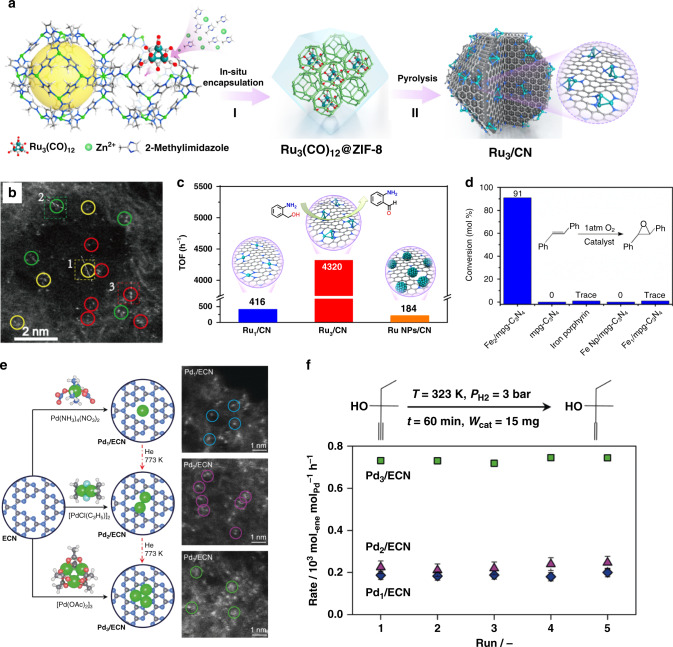
The precursor-preselected strategy. **a** Illustration of the Ru_3_/CN preparation process. **b** AC-HAADF-STEM images of Ru_3_/CN. **c** TOFs of the selective oxidation of 2-aminobenzaldehyde over the Ru_3_/CN, Ru_1_/CN, and Ru NPs/CN catalysts, reprinted with permission from ref. ^18^. Copyright (2017) American Chemical Society. **d** Catalytic epoxidation of *trans*-stilbene over Fe_2_/mpg-C_3_N_4_ and other samples^27^. **e** Approaches to prepare low-nuclearity Pd catalysts based on carbon nitride and the corresponding AC-HAADF-STEM images of the resulting catalysts. Broken red arrows indicate unsuccessful routes. Selected Pd atoms (blue circles), dimers (pink circles), and trimers (green circles) are identified. **f** Rates of the hydrogenation reaction of 2-methyl-3-butyn-2-ol during five runs. Adapted and reprinted with permission from ref. ^4^. Copyright (2019) John Wiley & Sons, Inc.

Metal–organic frameworks (MOFs) with a porous structure can spatially confine metal complex and serve as tunable support for atomic clusters. As in the case of MOFs, to encapsulate and confine the metal cluster precursors, the size of the cages in MOFs should be suitable. The diameter of a metal cluster precursor is ideal between one of the pores and cages. As a sub-family of MOFs, zeolitic imidazolate frameworks (ZIFs) are prepared by the self-assembly method containing M–Im–M structures (where M stands for Zn, Co, Cu cations and Im stands for imidazolate linkers). As shown in Fig. 4a, Wang et al. encapsulated Ru_3_(CO)_12_ into the cages of ZIFs, followed by pyrolysis for forming Ru_3_ clusters stabilized on nitrogen-doped porous carbon (Ru_3_/CN)^18^. The Ru_3_ clusters were identified by aberration-corrected high-angle annular dark-field scanning transmission electron microscopy (AC-HAADF-STEM, Fig. 4b). For the oxidation of 2-aminobenzaldehyde, Ru_3_/CN showed one order of magnitude higher TOF than the Ru_1_/CN and Ru NPs (Fig. 4c). Theoretical calculations revealed that the distinct catalytic activity linked to adsorption energies and adsorption configurations of the 2-aminobenzyl alcohol reactant on the three systems. When the Ru_3_(CO)_12_/ZIF-8 were reduced by hydrogen at a lower temperature, Ru_3_ clusters stabilized by ZIF-8 (Ru_3_@ZIF-8) were prepared due to the exceptional thermal stability of ZIF-8^83^. In the semi-hydrogenation of terminal alkynes, Ru_3_@ZIF-8 exhibited higher activity and selectivity than Ru_1_@ZIF-8 and Ru NPs@ZIF-8.

In addition, carbon nitride (C_3_N_4_) with abundant N sites can be employed to coordinate and stabilize the atomic metal clusters. For the synthesis Fe_2_ clusters (Fe_2_/mpg-C_3_N_4_), bis(dicarbonyl cyclopentadienyl iron) (Fe_2_O_4_C_14_H_10_) containing two Fe atoms in each molecule was selected as the precursor, and mesoporous C_3_N_4_ support was employed to anchor the Fe_2_ species^27^. The Fe_2_/mpg-C_3_N_4_ exhibited outstanding catalytic performance for epoxidation of *trans*-stilbene (Fig. 4d). DFT calculations revealed that neither too strong (on Fe nanoparticles) nor too weak (on iron porphyrin) Fe–O interactions could result in excellent catalytic activities.

In the case of Pd_6_/CeO_2_, Wang et al. selected Pd_6_L_4_ supramolecule (L = 1,3,5-tris(4-pyridyl)-2,4,6-triazine) as the precursor of Pd_6_ clusters^84^. For the oxidation of benzyl alcohols, Pd_1_/CeO_2_ showed extremely high activity, while Pd_6_/CeO_2_ was inert. DFT calculations revealed that owing to the larger size of Pd_6_ than Pd_1_, the Pd_6_ clusters impeded the interaction between hydroxyl groups of benzyl alcohols and CeO_2_ support, thus inhibiting the synergistic catalysis effect of Pd species and CeO_2_. These reports indicate the diversity of the nuclearity effect. The presence of neighboring metal atoms plays a vital role in the regulation of electronic structure. For some reactions, the regulation may lead to a positive change of the catalytic performance, but for other reactions, the change may be negative.

In addition, as shown in Fig. 4e, Pérez-Ramírez et al. fabricated Pd_1_, Pd_2_, and Pd_3_ clusters on C_3_N_4_ by using Pd(NH_3_)_4_(NO_3_)_2_, [PdCl(C_3_H_5_)]_2_, and [Pd(OAc)_2_]_3_ as precursors, respectively^4^. They tested two kinds of reactions, including semi-hydrogenation of alkynes and Suzuki coupling, and found application-dependent nuclearity effects. For the selective hydrogenation of various functionalized alkynes, Pd_3_/C_3_N_4_ was more active than Pd_1_/C_3_N_4_ and Pd_2_/C_3_N_4_ due to the reduced hydrogen activation barrier (Fig. 4f). In contrast, Pd_1_/C_3_N_4_ surpass Pd supported atomic clusters in Suzuki coupling, exhibiting distinct chemoselectivity and high stability.

The examples listed above demonstrate the general applicability of precursor-preselected strategy to synthesize supported atomic clusters. As mentioned above, the most critical point for this strategy is the selection of metal precursors and proper support. So far, porous materials, such as MOFs and C_3_N_4_, and oxides, such as CeO_2_, are candidate supports. We note that supported atomic clusters prepared by this strategy showed application-dependent nuclearity effects on the catalytic performances under realistic conditions. Since too weak interaction between the SACs and reactants usually leads to a high activation barrier, whereas too strong interaction between the nanoparticles and products blocks catalytic sites, both of which impair catalytic properties. On specific support, we can obtain intermediate interaction strengths by manipulating the nuclearity of supported atomic clusters and improve catalytic performances.

## The host–guest strategy

**Fig. 5:**
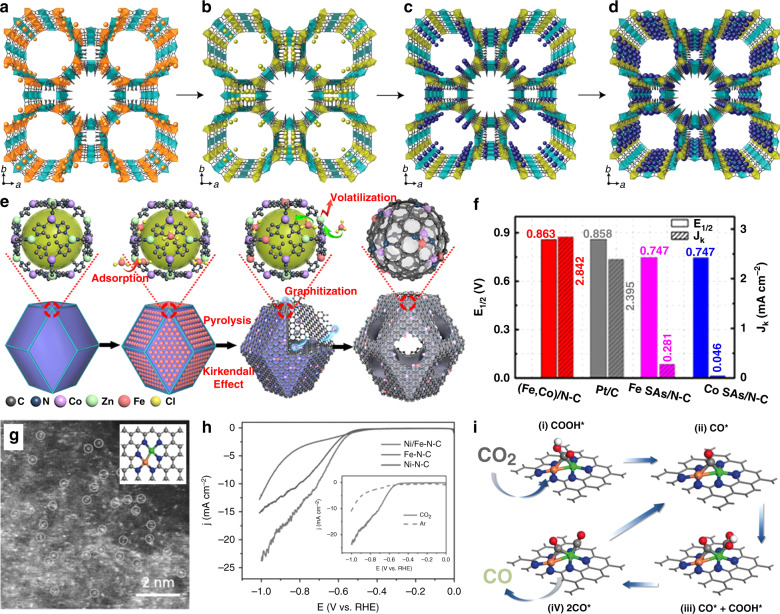
The host–guest strategy. **a**–**d** Design approach showing the structures of **1**–**4** determined by single-crystal X-ray diffraction from the three-step post-synthetic process consisting of a transmetallation **1** (**a**) to give **2** (**b**), an exchange of the Ni^II^ cations of the pores by [Pd^II^(NH_3_)_4_]^2+^ ones yielding **3** (**c**) and the final reduction process affording **4** (**d**). Reprinted by permission from Macmillan Publishers Limited: Springer Nature, *Nature Materials*, ref. ^85^ (Fortea-Pérez et al). Copyright (2017). **e** Preparation of (Fe, Co)/N–C. **f** Comparison of E_1/2_ and J_k_ for (Fe, Co)/N–C and other samples for ORR. Reprinted by permission from ref. ^19^. Copyright (2017) American Chemical Society. **g** AC-HAADF-STEM images of Ni/Fe–N–C. The inset of (**g**) is the structure model of Ni–Fe dual atoms (Fe: orange; Ni: green; N: blue; C: gray). **h** Linear sweep voltammetry (LSV) curves for CO_2_ reduction over Ni/Fe–N–C and other samples. Inset: LSV comparison for Ni/FeN–C in Ar- and CO_2_-saturated 0.5 M KHCO_3_ solution. **i** The catalytic mechanism on Ni/Fe–N site (based on the optimized structures of adsorbed intermediates COOH* and CO*). Adapted and reprinted with permission from ref. ^28^. Copyright (2019) John Wiley & Sons, Inc. (Wiley).

The host–guest strategy based on reduction is advantageous in preparing supported atomic clusters, especially nitrogen-coordinated dual-metal sites that contain heteroatoms. MOFs, a type of porous materials, are excellent platforms to confine and prepare atomic clusters in a controlled way. Pardo et al. synthesized the Pd_4_ clusters stabilized by MOF (Pd_4_-MOF) through a three-step post-synthetic process (see Fig. 5a–d)^85^. In this process, initial MOF containing Mg^2+^ and Cu^2+^ cations were converted to a more robust MOF containing Ni^2+^ and Cu^2+^ cations by a transmetallation step; then partial Ni^2+^ cations were exchanged by [Pd(NH_3_)_4_]^2+^, and NaBH_4_ was used as the reductant to give the final compound Pd_4_-MOF. Substantial evidence for the structures of MOFs in different stages came from single-crystal X-ray diffraction measurement. The quasi-linear Pd_4_ clusters were stabilized by a synergic effect between the MOF network and the solvent molecules. The MOF-supported Pd_4_ atomic clusters outperformed state-of-the-art metal catalysts in carbene-mediated reactions of diazoacetates with high activity (yield > 90%). Also, the Pd_4_ atomic clusters retained their catalytic activity in the flow reactions (>20 cycles). Whereas a series of Pd-based salts, coordination compounds, and commercial Pd nanoparticles on different supports showed much lower catalytic activities (yield < 34%) under the same conditions. In the next year, Pt_2_-MOF was synthesized via a similar process by the same Pardo group^86^. The specially designed MOFs are critical for the generation of the Pd_4_ and Pt_2_ clusters.

In 2017, Wu et al. used Co-doped ZIF-8 as the host and encapsulated Fe^3+^ within the cavities (see Fig. 5e)^19^. During the pyrolysis process, Zn^2+^, Co^2+^, and Fe^3+^ ions were reduced by as-generated carbon, and Zn atoms were removed subsequently. The existence of dual-metal sites was proved by the AC-HAADF-STEM image. Combining XAFS and Mössbauer spectroscopic analysis, the presence of the Fe–Co bond was confirmed. For the acidic ORR, the dual Fe–Co sites supported on N-doped porous carbon showed higher activity than both Fe- and Co-SACs (see Fig. 5f). At the same time, the dual-metal catalysts exhibited comparable onset potential and half-wave potential with commercial Pt/C. Also, the dual-metal catalysts were stable in an accelerated durability test with 50,000 cycles. DFT calculations reveal that the reduced activation barrier of the O–O bond on dual-sites is crucial for the process of four-electron oxygen reduction. Recently, Wu et al. found the Fe–Co dual-metal catalysts could catalyze CO oxidation with 100% conversion at a low temperature (−73 °C), whereas the Co-SACs and Fe SACs showed CO conversions below 15% and 0, respectively^87^. XANES spectra, pulse-adsorption microcalorimetry, and DFT studies showed that the Fe–Co dual-metal sites catalyzed CO oxidation synergistically, with CO and O_2_ preferentially adsorbing at the Co and Fe sites, respectively. Very recently, Xing et al. also studied the enhanced ORR performance of Fe–Co dual-atom sites. The Fe–Co catalyst was also prepared by pyrolyzing Fe-impregnated ZnCo-ZIF^88^.

Inspired by these works, Ni–Fe dual-metal sites were successfully prepared by using Fe-doped ZIF-8 and Ni(NO_3_)_2_ as the host and guest, respectively^28^. XAFS and DFT simulations confirmed the existence of Ni–Fe coordination. The Ni–Fe dual-metal catalyst prepared by pyrolysis also shows enhanced activity than the corresponding SACs (Ni–N–C and Fe–N–C) for CO_2_RR (see Fig. 5g, h). The Ni–Fe–N–C exhibits a maximum FE_*CO*_ of 98% at −0.7 V. Besides high activity, this dual-metal catalyst also displays outstanding stability for CO_2_RR, maintaining high FE_*CO*_ of 99% during 30 h of continuous electrolysis. DFT studies indicate that the bimetal-nitrogen sites undergo a geometric change into a CO-adsorbed moiety upon CO_2_ adsorption, which decreases the energy barrier for the formation of COOH* and desorption of CO (see Fig. 5i).

Metallic bonding emerges when atomic orbitals on adjacent atoms overlap substantially. Supported atomic clusters containing several kinds of metal atoms (hetero-ACs) provide more opportunities for the manipulation of electronic structure than that containing only one kind of atom. At the same time, inter-cluster polarization and atomic interface effects in hetero-ACs may create remarkable catalytic properties. Upon different reagent molecules co-adsorbed on adjacent and different kinds of metal sites, the reaction barrier can be decreased, leading to exceptional high catalytic activity. The host–guest strategy is powerful to prepare hetero-ACs, and plenty of works are worth trying.

## The wet chemical reduction method

**Fig. 6:**
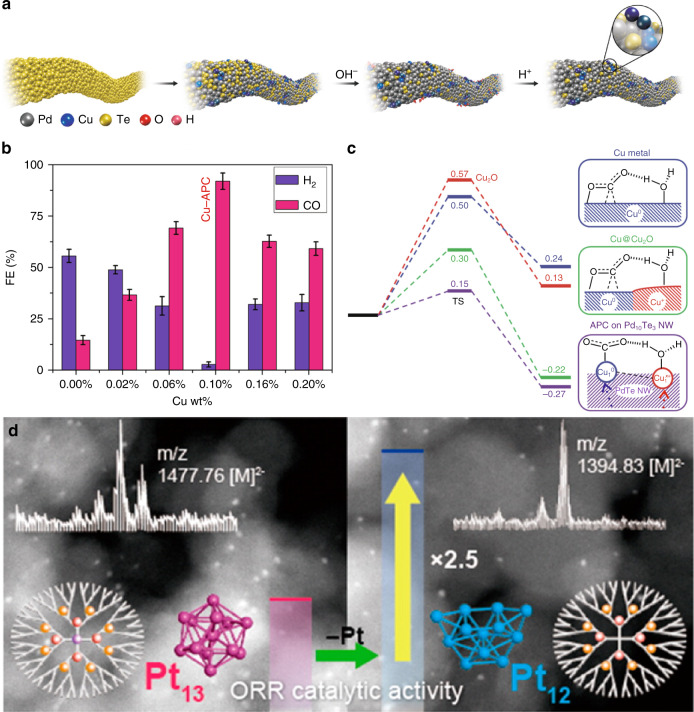
The wet chemical reduction and dendrimer-based strategies. **a** Schematic illustration of the synthesis of atom-pair structured Cu anchored on Pd_10_Te_3_ nanowires. **b** FE_CO_ and FE_H2_ of samples with different Cu loading at −0.78 V (versus RHE) in CO_2_RR. **c** Free energy profiles for CO_2_ activation on Cu, Cu@Cu_2_O, and APC of Cu_1_^0^–Cu_1_^x+^ on Pd_10_Te_3_ nanowires. Reprinted by permission from Springer Nature Limited: Springer Nature, *Nature Chemistry*, ref. ^30^ (Jiao et al). Copyright (2019). **d** HAADF-STEM images and ORR catalytic activity of Pt_13_ (left) and Pt_12_ (right) clusters supported on mesoporous carbon. Reprinted by permission from ref. ^2^. Copyright (2013) American Chemical Society.

Wet chemical reduction method was employed to prepare supported atomic clusters in which metal nanomaterials behaved as the supports for the clusters. Chen et al. used the Te nanowires as partial sacrificial templates to obtain Cu-loaded (Cu_4_ clusters) PdTe nanowires (see Fig. 6a)^30^. NaOH and HCl solutions were used to wash the solid product in sequence. On this nanowires, stable Cu_1_^0^–Cu_1_^x+^ pair in Cu_4_ clusters was the essential catalytic active site for the electrochemical reduction of CO_2_, which was proved by DFT calculation and experimental and fitting XAFS spectroscopy. The stable Cu_1_^0^–Cu_1_^x+^ pair in Cu_4_ clusters can selectively catalyze the CO_2_RR (see Fig. 6b). The Faradaic efficiency for CO (FE_*CO*_) is above 92% under a relatively low potential (−0.78 V). Theoretical calculations reveal that the Cu_1_^0^–Cu_1_^x+^ pair directly participate in the CO_2_RR. At the same time, the other two Cu atoms in the Cu_4_ clusters stabilize the Cu clusters (see Fig. 6c). According to the theoretical calculations, Chen et al. have predicted stable Cu dimer supported by C_2_N can exhibit high selectivity for CH_4_ with a small limiting potential (−0.23 V). They also forecast C_2_H_4_ can also be produced on the Cu_2_@C_2_N^89^. Different pathways take place for the formation of CO, formic acid, hydrocarbons (C_2+_), and multicarbon oxygenates. The C–C coupling step is vital for the production of the latter two kinds of species.

Chen et al. prepared Pt_3_ decorated Co@Pd nanocatalyst by reducing Co, Pd, and Pt in sequence^31^. In this method, the short reduction time of Pt (10 s) was vital for the formation of Pt_3_ clusters. Since the theoretical average coordination number of Pt–Pt bond was 2.0, and the fitting XAFS data of this Pt decorated nanocatalyst was 1.95, indicating the presence of Pt_3_ clusters. Compared with carbon supports, the metal nanomaterials show much brighter contrast in HAADF-STEM images, and it is difficult to identify the metal clusters via this visible characterization technique. For ORR in alkaline aqueous solution, the unique charge localization induced by Pt_3_ decoration on Co@Pd nanocatalyst resulted in a distinct mass activity, which is more than 30 times higher than that of commercial Pt/C.

The wet chemical reduction method is practical for the production of industrial-scale quantities of catalysts. However, the clusters prepared by this method usually have a broad size range. Capping ligands on nanostructures play a vital role in controlling morphology and prevent agglomeration. In some instances, the ligands are likely to impact the oxidation states of the clusters and the access of the reagent molecules. Therefore, there are limitations to the precise synthesis of supported atomic clusters by wet chemical reduction method.

## The dendrimer-based strategy

N-doped carbon materials are widely used to support and stabilize the atomic clusters via ligand protection from N coordinations. Dendrimers, macromolecules with a tree-like structure, contain a high proportion of N. At the same time, they have well-defined structures, as analogous to MOFs. Before MOFs, the dendrimers behaved as the nanosized reactor in which to synthesize various kinds of supported atomic clusters^51,90–92^. Dickson et al. reported the synthesis of Ag_n_ clusters using a PAMAM G2 dendrimer (second generation of poly-(amidoamine) dendrimer) by a photochemical reduction method^32^. However, the clusters prepared were determined to be a mixture of Ag_2_, Ag_3_, and Ag_4_ by electrospray ionization mass spectrum (ESI-MS). When they reduced Au ions in PAMAM by using a chemical reducing agent (NaBH_4_), Au nanoparticles and Au_8_ clusters were prepared. They removed larger Au nanoparticles through centrifugation^90^. It is interesting to note that only the Au_8_ cluster encapsulated by PAMAM was observed by ESI-MS. This work is a breakthrough for the synthesis of a metallic cluster with an atomic precise in the solution phase.

Besides PAMAM, scientists also prepared metal clusters using DPA (dendritic phenyl azomethine)^92,93^ and PPI (polypropylene imine)^94^ dendrimers as host. Taking advantage of the basicity gradient of DPA dendrimer, scientists can prepare multimetallic nanoclusters precisely^95,96^. The supported atomic clusters prepared by the chemical dendrimer-based method also show size-dependent catalytic performance. Yamamoto et al. stabilized the Pt_12_ and Pt_13_ clusters by the DPA-TPM (fourth-generation DPA with a tetraphenylmethane core) and DPA-PyTPM (fourth-generation DPA with a triphenylpyridylmethane core), respectively. For acidic ORR, the Pt_12_ atomic clusters showed more than twofold mass activity compared with that of the Pt_13_ atomic clusters (see Fig. 6d). DFT analyses suggested that the different atomic coordination of Pt_12_ (deformed) and Pt_13_ (icosahedral) caused different catalytic activity^2^. Thus, by influencing the atomic coordination, the metal nuclearity can determine the catalytic performance significantly.

It should be noted that different dendrimers showed different capacities of encapsulation and stabilization, which influenced the catalytic performance consequently^97^. Due to the limited restriction capacity of dendrimers, the nuclearity of supported atomic clusters prepared by this method always larger than 10^33^. As a result, the studies for the catalytic performance of supported atomic clusters with nuclearity smaller than 10 are few. On the other hand, taking advantage of phenyl azomethine dendrimer’s basicity gradient, scientists can precisely prepare multimetallic nanoclusters.^95^ However, the dendrimers used in these reports are mainly synthesized in the lab, limiting the scalable production of the atomic cluster by this method.

## The atomic layer deposition method

**Fig. 7:**
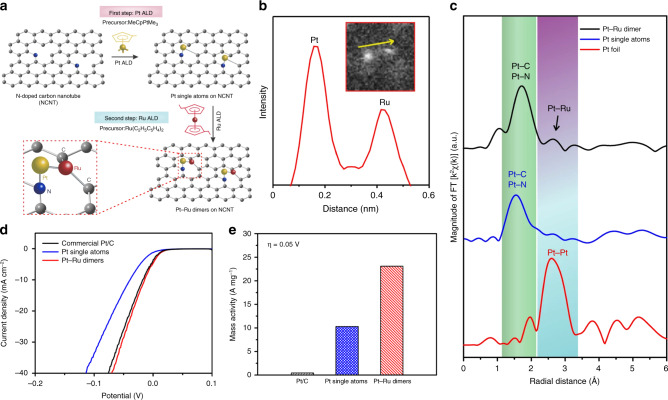
The atomic layer deposition method. **a** Schematic illustration of the synthesis of Pt–Ru dimers on nitrogen-doped carbon nanotubes. **b** The intensity profile of one individual Pt–Ru dimer from the enlarged AC-HAADF-STEM image of inset. **c** Fourier transforms of the Pt EXAFS spectra for the Pt–Ru dimers, Pt single atoms, and Pt foil. **d**, **e** Electrocatalytic HER performance of Pt–Ru dimers and other samples. The polarization curves (**d**) and normalized mass activity at 0.05 V (**e**) of Pt–Ru dimers, Pt single atoms, and Pt/C catalysts^34^.

The atomic layer deposition (ALD) method is robust in preparing M_2_ atomic clusters supported on the defect-rich carbon materials^3,34^. Very recently, Sun et al. reported the synthesis of one-to-one Pt–Ru dimers supported on N-doped carbon nanotubes (NCNT) via a two-step ALD process (see Fig. 7a)^34^. They studied the depositions of Pt and Ru on NCNT, respectively, and found the easier deposition of Pt than Ru. So they firstly prepared isolated Pt atoms on the NCNT support by exposing NCNT with trimethyl(methylcyclopentadienyl)platinum(IV) (MeCpPtMe_3_). Utilizing the different interactions between the Ru precursors and Pt or NCNT, they could deposit Ru atoms on Pt atoms selectively in the second step. In the AC-HAADF-STEM image, Pt atom was brighter than the Ru atom (see Fig. 7b). As depicted in the Fourier transforms of the Pt EXAFS spectra (see Fig. 7c), there was a main Pt–C/N bond peak (1.6 Å) and a small Pt–Ru bond peak (2.6 Å) in the Pt–Ru dimer catalysts. Compared with the Pt SACs, the Pt–Ru dimers exhibited better activity for electrochemical hydrogen evolution reaction (HER). Besides, the Pt–Ru dimers showed much higher mass activity (54 times) and excellent stability compared to commercial Pt/C catalysts (see Fig. 7d, e). Based on the DFT calculations, when three H atoms adsorbed on both Pt and Ru sides of the Pt–Ru dimer, the corresponding Gibbs free energy for Pt(3H)Ru(3H) → Pt(3H)Ru(2H) was only 0.01 eV, which was smaller than that of Pt SACs (0.1 eV) and Pt–Pt dimer catalyst (−0.14 eV). In 2017, Lu et al. have used the same precursor to prepare Pt dimers on graphene by the ALD method. However, they proposed that the Pt dimers were in the oxidized form of Pt_2_O_x_^3^.

In the works as mentioned earlier, the crucial factors in preparing isolated dimers are mainly two interactions: the steric hindrance between precursor molecules and the self-limiting reactions between precursor molecules and the supports. Therefore, the choice of the metallic precursor with huge ligands, such as methylcyclopentadienyl, is very fundamental. However, the high cost of these kinds of precursors hampers the practical application of the ALD method. All in all, this ALD method can prepare monometallic and bimetallic atomic clusters precisely, but the limited output in the sophisticated equipment and expensive precursors restrict the industrial scalability.

## Other methods

Prof. Christopher et al. have developed strong electrostatic adsorption (SEA) method to deposit atomically dispersed heteroatom species consisting of late-transition metals on metallic oxides. Besides the weight loading, solution volume, and solution pH, the precursors’ choice is also significant. The surface oxygen species will change from hydroxyl (OH) to oxygen anion (O^−^) when the solution changes from neutral to alkaline^98^. By choosing a cationic precursor (always coordinated by four ammine molecules), the Coulombic attraction between the surface O^−^ and the precursor promotes the deposition. Using this SEA method, they prepared Pt_1_/CeO_2_^99^, Pt_1_/TiO_2_^100^, and Rh-ReO_x_/Al_2_O_3_^101^ atomically dispersed catalysts.

Prof. Notestein et al. have developed the vapor-phase metalation by atomic layer deposition (ALD) in a metal–organic framework (MOF), during which the temperature is always in the range of 110–160 °C. The key to this strategy is the synthesis of NU-1000, a thermally stable, Zr-based MOF^102^. Various classes of volatile metal precursors containing alkyl, alkylamide, amidinate, carbonyl, and metallocene have been used to postmodify the NU-1000^102–106^. NU-1000 has 3 and 0.8 nm pores, and the deposited metal species mainly occupy the small pores centered between the nodes. Their loading is always smaller than eight metals/Zr_6_ nodes.

Prof. Lopez-Quintela et al. have developed an electrochemical method to prepare metallic clusters with precise number^107^. Naked Ag_2_ and Ag_3_ clusters can be prepared using a very low concentration of Ag ions in solution^108^. Combing with DNA-acting drugs, the Ag_3_ clusters that strongly intercalate with DNA play an essential role in improving cytotoxicity to human lung carcinoma cells^109^. Using a modified electrochemical method, Au_3_, Au_5–7_, and Au_7–10_ clusters were synthesized and supported on functionalized carbon nanotubes by a wet impregnation procedure. In the oxidation of thiophenol with O_2_, the single Au atoms are not active, while Au_5–10_ clusters are very active^110^.

## Outlook

The development of synthesis methods is of great significance for the practical application of supported atomic clusters with well-defined structures. The synthesis of supported atomic clusters has made significant progress since early preparation in UHV conditions. In this perspective, six strategies have been summarized and classified for preparing supported atomic clusters, including gas-phase synthesis and size-selected strategy, precursor-preselected strategy, host–guest strategy, wet chemical reduction method, dendrimer-based strategy, and atomic layer deposition. In terms of precision synthesis, multilateral clusters, and industrial scalability, we have compared the advantages and disadvantages of each strategy and summarized them in Table 1.

**Table 1:** Comparison of six synthesis strategies in terms of precision synthesis, multilateral clusters, and industrial scalability.

Synthetic strategies	Precise syntheesis	Multimetal clusters	Industrial scalability
Size-selected strategy	Suitable	Suitable	Not suitable
Precursor-preselected strategy	Suitable	Suitable	Suitable
Host–guest strategy	Suitable	Suitable	Suitable
Wet chemical reduction strategy	Not suitable	Suitable	Suitable
Dendrimer-based strategy	Not suitable for M_n_ (*n* < 10)	Suitable	Not suitable
Atomic layer deposition strategy	Suitable	Suitable	Not suitable

We also highlight the catalyst application of supported atomic clusters and extend a discussion to the structure-activity relationship of them through the specific instances. With the in-depth development of the researcher’s cognition and the continuous progress of science and technology, the synthesis and application of supported atomic clusters in catalysis will be full of new opportunities but remain grand challenges.

## The synthesis of supported atomic clusters

A critical restriction in the development of supported atomic clusters is the lack of general methods to efficiently and precisely access supported atomic clusters with high performance on a large scale. Meanwhile, it is still challenging to achieve a controllable synthesis of supported atomic clusters with precise atom numbers. Up to now, most of the reported M_2–10_ supported atomic clusters have been prepared by chance. Thus, there is not systematic and practical guidance for the synthesis of supported atomic clusters. If we want to synthesize supported atomic clusters with precise atoms in a targeted way by chemical methods, we had better have a deeper understanding of the critical factors to obtain stable structures. For example, the competitive binding between metal–support and metal–metal determines the stability of corresponding SACs. When the former is thermodynamically stable than the latter, it may be a driving force for the transformation of SAC from nanoparticles. Whether the experimental conditions can provide enough energy for the kinetic barrier of the transformation is another crucial facet. Therefore, employing systematic experiments and theoretical calculations, we may be able to predict the more stable configuration of single-cluster catalysts anchored on different supports, which is crucial for the synthesis of supported atomic clusters.

To develop the synthesis of supported metallic atomic clusters, following six factor can be considered. (1) The bottom-up strategy to prepare supported atomic clusters should be given enough attention, during which the monoatomic precursors are absorbed, assembled, reduced, and confined by the defects of the supports. (2) The recently developed matrix assembly cluster source (MACS)^111^ and high-power impulse magnetron sputtering (HiPIMS)^112^ methods present a bridge between the laboratory research scale and the industrial levels. The former method produces cluster-decorated powder catalysts at the gram scale. (3) The production rates of simple nanoparticles in conventional aerosol flame technology reach 25 t/h, so it may be extended to produce supported atomic clusters in large scale, during which the main challenge is the precise control of the atomicity^113^. (4) The top-down strategy, based on the decomposing of ordered nanostructures into smaller species, is worth a test, which is proven effective in the scalable synthesis of SACs. (5) The strategy for precise handling of the nuclearity of supported atomic clusters by the wet chemical method should be developed to enrich the variety and obtain multifunctional materials. (6) The manipulation of the electronic structures of supported atomic clusters may be achieved by tuning the geometric structure of atomic clusters, or by changing the chemical environment of the supports.

## The characterization of supported atomic clusters

As we know, AC-HAADF-STEM analysis usually represents the direct two-dimensional projection of the supported atomic clusters samples along the direction of the incident beam. Thus, the observed atomic structure images cannot fully reflect the three-dimensional perspective of the supported atomic clusters samples. Besides, the thickness of an as-prepared catalyst in the z-axis is always tens or hundreds of nanometers, which may make several single-cluster sites coincide in the direction of the incident beam. In particular, when the supported atomic clusters contain multiple metal atoms with similar ordinal numbers, such as iron, cobalt, and nickel, they will display similar contrast in HAADF-STEM images, which will make it more difficult to identify them from each other. Therefore, there are many difficulties in the unambiguous characterization of supported atomic clusters.

The X-ray absorption fine spectrum (XAFS) is a powerful tool to analyze the chemical environment and coordination structure of the supported atomic clusters. It is not like the SACs system that no metal–metal bond can strongly prove the atomic dispersion of metal species. For the supported atomic clusters, it is not only necessary to confirm that there are no nanoparticles, but also to prove that there are limited metal–metal bonds among metal cluster atoms. Also, the single clusters supported on host materials doped by various kinds of light atoms (C, O, N, P, S, etc.), the atomic structures of the supported atomic clusters are too complex to be determined, owing to a variety of possible structural coordination between metals and light atoms. Notably, the identification of dimers and trimers supported on the same material is much more difficult. To make the characterization easier, researchers can employ ultrathin or 2D supports for preparing the supported atomic clusters. With the advances of characterization, the 3D reconstruction technique may provide a possible way to confirm the more stereoscopic or realistic atom structure for supported atomic clusters. Combining with the theoretical calculation, we can provide an effective way for the investigation of supported atomic clusters.

## The catalytic study of supported atomic clusters

The development of a synthesis strategy also promotes the investigation of catalytic systems under actual reaction conditions. In the reported papers, the performances of SACs are sometimes better than these of the supported atomic clusters, on the contrary, it often appears, and there is a lack of disciplinary and systematic understanding of supported atomic clusters in catalysis. The essential reason is derived from the uncontrollable design in synthesis. For example, the system of Pd_1_ and Pd_6_ reported in the literature is also an unexpected finding. We can attempt to design and synthesize the realistic Pd_1–10_ materials and systematically study their differences in structure and performance, which will provide an experimental basis for the study of real active species and the design of optimized catalysts. In addition, the strong interaction between metals and supports affects the geometry and stability of the supported atomic clusters, owing to the charge transfer between supports and clusters. Therefore, it is one of the essential factors in the study of the catalytic applications of supported atomic clusters.

Five aspects should also be given enough attention. (1) The dynamic behavior of the supported atomic clusters during catalytic processes. In situ spectroscopic (X-ray absorption spectroscopy, XPS, X-ray diffraction) and microscopic (TEM) techniques that allow tracking the transformation during the catalytic process will be particularly helpful. (2) The non-uniformity of the support materials denotes that not all the single-cluster centers in supported atomic clusters are equally active. This non-uniformity may bring about the diversity of the catalytic performances, including the activity, selectivity, and stability. (3) Heterogeneous supported atomic clusters can catalyze the homogeneous reactions with distinct yield and selectivity. (4) Based on the scalable synthesis of supported atomic clusters, we should promote the application of supported atomic clusters in the industrial field. (5) Concomitant advances in synthesis, characterization, and development in molecular modeling, have been beneficial in understanding the structure–function relationships of supported atomic clusters, as well as to predict their catalytic performance. For a target reaction, a specific catalyst will be designed at the atomic and molecular scales, and the green, atomic economy catalytic processes will be realized.
